# Bacterial Communities in Various Parts of Air-Conditioning Units in 17 Japanese Houses

**DOI:** 10.3390/microorganisms10112246

**Published:** 2022-11-13

**Authors:** Kensuke Watanabe, U Yanagi, Yoshiki Shiraishi, Kazuhiro Harada, Fumitoshi Ogino, Koichiro Asano

**Affiliations:** 1 Graduate School of Engineering, Kogakuin University, Tokyo 163 8677, Japan; 2 School of Architecture, Kogakuin University, Tokyo 163 8677, Japan; 3 Division of Pulmonary Medicine, Department of Medicine, Tokai University School of Medicine, Isehara 259 1193, Japan; 4 Research & Development, Duskin Co., Ltd., Osaka 564 0043, Japan

**Keywords:** residential building, air conditioner, air filter, cooling coil, fan, air outlet, bacteria, next-generation sequencing, 16S rRNA gene

## Abstract

HVAC systems have a significant impact on the indoor environment, and microbial contamination in HVAC systems has a significant effect on the indoor air quality. In this study, to gain a better understanding of the microbial contamination inside ACs, we used NGS to analyze the 16S rRNA gene of bacteria adhering to AC filters, cooling coils, fans, and air outlet surfaces. The five phyla in terms of the highest relative abundance were *Proteobacteria*, *Firmicutes*, *Actinobacteria*, *Cyanobacteria*, and *Bacteroidetes*. The surface of an AC filter provides a history of indoor airborne bacterial contamination, and of the 10 bacterial genera we detected with the highest abundance (in the following order: *Pseudomonas* > *Staphylococcus* > *Paracoccus* > *Corynebacterium* > *Acinetobacter* > *Streptococcus* > *Methylobacterium* > *Enhydrobacter* > *Sphingomonas* > *Actinotignum*) on the filter surface, the top 6 genera were Gram-negative bacteria. Furthermore, the seventh-most abundant genus adhering to the filter surface (*Methylobacterium*) was the second-most abundant genus on the cooling coil and fan, and the ninth-most abundant genus on the air filter (*Sphingomonas*) was the third-most abundant genus on the cooling coil. Various factors impact the bacterial flora inside AC units, including the location of the house, AC unit usage, and occupant activity.

## 1. Introduction

In developed countries, people spend around 90% of their time indoors [1]. The indoor environment plays host to a variety of microbes, such as bacteria, fungi, and viruses [2,3], and includes a wide variety of bacterial sources [4], such as the outside air [5], occupants [5,6,7,8,9,10,11], pets [12,13], houseplants [14,15], and air-conditioning (AC) systems [7,16,17,18,19,20,21,22,23,24]. The heating, ventilation, and air-conditioning (HVAC) systems have a significant impact on the indoor environment, and microbial contamination in HVAC systems has a significant effect on indoor air quality.

Air conditioning is common in developed countries. In Japan, AC units are installed in more than 90% of the homes [25], and microbial contamination inside AC systems is an important factor in people’s health. Banaszak et al. [26], Baur et al. [27], and Acierno et al. [28] have reported that *thermophilic actinomycetes* growing inside humidifiers and AC units cause hypersensitivity pneumonia. There are also reports that endotoxins, a component of the cell wall in Gram-negative bacteria, may be associated with allergic sensitization in humans [29,30] and exposure to airborne endotoxins is associated with workplace-related illnesses [31,32,33].

When an AC unit operates in cooling mode, the cooling coil inside the AC unit works to cool and dehumidify the air in the room. To achieve this, condensation forms on the cooling coil and a high-humidity environment is created inside the AC unit. Given a high-humidity environment, bacteria will proliferate on a wide variety of component surfaces. Dannemiller et al. used glass chambers to investigate the effect of relative humidity on microbial growth in floor dust and observed fungal growth after 1 week at 90% relative humidity and bacterial growth after 1 week at 100% relative humidity [34]. Hyvärinen et al. also investigated microbial growth in actual indoor environments on seven different moisture-damaged building materials (wood, paper, non-wooden building boards, ceramic products, mineral-based insulation materials, paints and glues, and plastics) and observed bacterial growth on all materials but particularly pronounced growth on insulation materials [35]. Not only is bacterial proliferation on cooling coils an important factor in the health of occupants, but bacteria can also form biofilms [18,19,22,36] that reduce the heat-exchange efficiency of the equipment.

Various published reports provide details of the phyla, genera, and species of bacteria that have been detected to date in HVAC systems. *Streptophyta* is the predominant phylum found adhering to HVAC system filters, a phenomenon reportedly affected by both indoor and outdoor air [7]. Using culture-based analysis, Hugenholtz et al. detected *Acinetobacter*, *Arthrobacter*, *Bacillus*, *Corynebacterium*, *Pseudomonas*, and *Staphylococcus* on air handling system coils [19]. Using next-generation sequencing (NGS), Bakker et al. identified *Methylobacteriaceae*, *Propionibacterium*, *Acetobacteraceae*, *Sphingomonas*, *Pseudanabaenaceae*, *Streptophyta*, *Acinetobacter*, *Bacillales*, *Hymenobacter*, and *Corynebacterium* as the predominant bacteria on AC unit coil surfaces [21]. Using NGS, Hatayama et al. identified the genus *Methylobacterium* and the family *Sphingomonadaceae* as the predominant bacteria on the evaporator of split-type air conditioners in Japanese homes [20].

Most of the studies published to date have focused on bacteria attached to the air filters and cooling coils of AC systems in commercial buildings. Although Hatayama et al. did publish a report on the bacteria found on coil surfaces in residential AC units [20], almost no other reports that focus on residential homes examine in detail the ductless mini-split-type AC units widely used in Japanese homes. Looking at the AC unit in terms of the airflow, the air filter, the coil, the fan, and the air outlet surface, each feature has different temperature and relative humidity conditions. Hence, each presents an environment suited to the proliferation of different types of bacteria. Understanding the bacterial flora on each of these features will help to determine the effects of the bacteria in air conditioners on indoor environments. Therefore, we examined the bacterial flora attached to filters, coils, fans, and air outlet surfaces of room air conditioners in 17 homes in Kanagawa prefecture, Japan, and investigated the characteristic features of the bacterial flora on each of these components.

## 2. Materials and Methods

### 2.1. Measured Locations

Measurements were performed in the living rooms of 17 homes in Kanagawa prefecture, Japan, between 3 July and 26 September 2021 (during summer). Table 1 provides an overview of the homes where measurements were taken, AC unit usage, and the number of occupants.

### 2.2. Temperature and Relative Humidity

**Figure 1:**
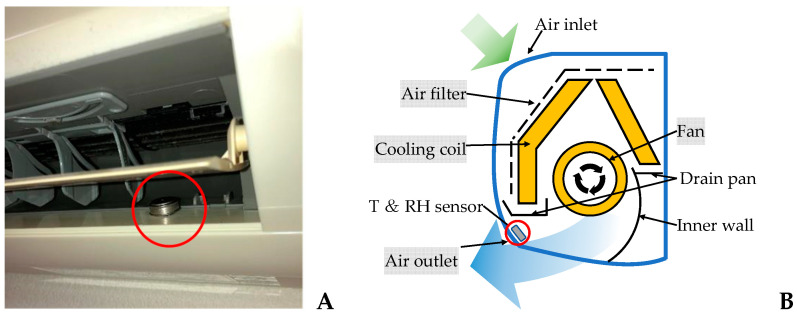
Location of sensors for measuring T and RH at air the conditioner air outlet (**A**), configuration of the ductless mini- split-type AC unit (**B**).

To understand the temperature (T) and relative humidity (RT) conditions inside an AC unit operating in cooling mode, temperature and relative humidity were measured continuously at 30-min intervals by a compact temperature/humidity sensor (Hygrochron, KN Lab Series). To represent the conditions at the air inlet of the AC unit, measurements were taken in indoor air. To represent the conditions at the cooling coil in the AC unit and the air downstream from the cooling coil, measurements were taken at the air outlet of the AC unit (Figure 1). To investigate the bacterial flora adhered to each component in the room AC unit, the bacteria were analyzed in order of airflow, i.e., bacteria adhered to the air filter, bacteria adhered to the cooling coil, bacteria adhered to the fan, and bacteria adhered to the air outlet surfaces. A sterile cotton swab was used to wipe bacteria from a 25 cm^2^ surface area on each component. The cotton swabs were then stored individually in a container (ST-25, ELMEX) containing 10 mL of sterile phosphate-buffered solution and taken to be processed in a laboratory. Samples were collected from two sites of each component, for a total sampled surface area of 50 cm^2^.

### 2.3. DNA Extraction, Amplification, and Sequencing

#### 2.3.1. DNA Extraction

After samples of adhered bacteria were collected as described above, the cotton swabs were processed with a stomacher (MiniMix 100 P CC Interscience), 3 mL of DNase-free water was combined with 2 mL of the sample solution, and the DNA was extracted with a Stomacher Biomaster device. The processed sample was then removed from the stomacher bag and placed in a 1.5 mL test tube and centrifuged (KUBO-TA5911) at 4 °C and 3000 rpm for 30 min to extract the bacteria. DNA was purified using a NucleoSpin® Tissue kit (740952, MACHEREY-NAGEL) and by mixing the process liquid in a vortex mixer, heating it, combining it with ethanol, performing centrifugal separation, and carrying out other process steps.

#### 2.3.2. DNA Amplification and Sequencing

For each sample, the variable region 4 (V4) of the bacterial 16S ribosomal RNA (rRNA) gene was amplified by polymerase chain reaction (PCR) using the primer “ACACTCTTTCCCTACACGACGCTCTTCCGATCT-GTGCCAGCMGCCGCGGTAA (1st_515F)” [37], and “GTGACTGGAGTTCAGACGTGTGCTCTTCCGATCT-GACTACHVGGGTWTCTAAT (1st_806R)”; “AATGATACGGCGACCACCGAGATCTACACxxxxxxxxACACTCTTTCCCTACACGACGC (2nd forward primer)”, and “CAAGCAGAAGACGGCATACGAGATxxxxxxxxGTGACTGGAGTTCAGACGTGTG (2nd reverse primer)”. DNA amplification and 16S rRNA gene analysis performed on Illumina NGS, the collected DNA was outsourced to a commercial laboratory.

#### 2.3.3. DNA Sequencing and Analysis

DNA quality was verified using an Agilent 2200 TapeStation, and all samples containing nucleic acid concentrations of the quality and quantity required for analysis were analyzed. The generated sequence libraries were combined, and the re-amplified PCR products were purified with AMPure XP beads (bead volume ratio 1:1) to improve the quality of the sequence libraries. Data were analyzed using QIIME (Ver.1.9.0, Silva 132 Database).

## 3. Results

### 3.1. Temperature and Humidity

**Figure 2:**
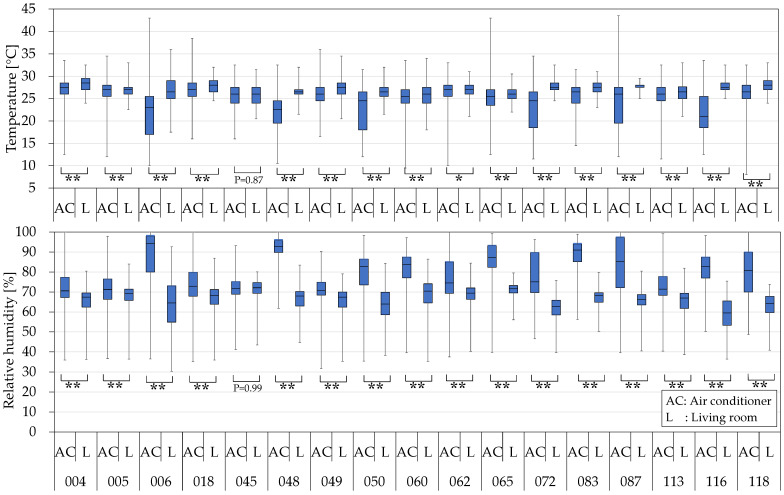
The quartile values of air temperature and relative humidity of living room and air conditioner air Outlet. Statistical significance was evaluated by Mann−Whitney U test. * *p* < 0.01, and ** *p* < 0.001.

During the cooling operation, the temperature at the air outlet of an air conditioner (AC) is lower than the living room temperature (L), and the relative humidity is higher. Statistical analysis using SPSS Statistics 29 revealed significant differences between the indoor and air outlet temperatures and relative humidity, except for house 045. (Figure 2). House 045 had the lowest air conditioning usage frequency (Table 1).

**Figure 3:**
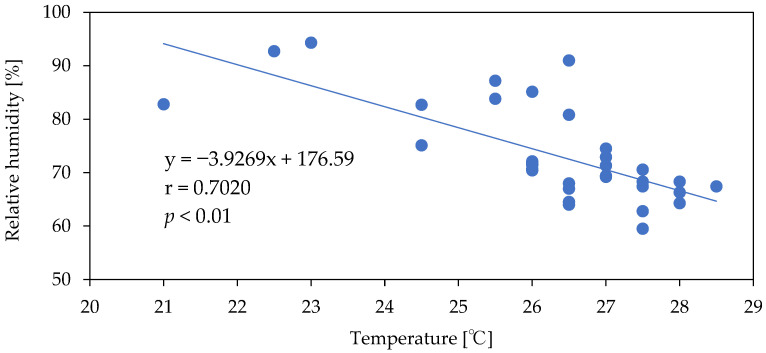
Relationship between median temperature and median relative humidity.

The temperature and relative humidity measurements show a large degree of variation because they were collected both while the AC unit was operating in cooling mode and while the AC unit was not in operation (Figure 2). Overall, the relative humidity tends to increase when the outlet temperature is low (Figure 3). In addition, because the temperature/humidity sensor at the air outlet is exposed to indoor air, when the AC unit is not in operation, the sensor is affected by the temperature and relative humidity in the room. This explains why house 045, which had the lowest frequency of AC unit usage (Table 1), produced almost identical 75th percentile, median, and 25th percentile results for the living room temperature measurements and the AC unit air-outlet-temperature measurements. The maximum temperature of the AC unit was higher than that of the living room due to the warm air produced when the AC unit operates in drying mode. In drying mode, a four-way valve in the AC unit refrigerant flowline temporarily reverses the flow of the refrigerant, the evaporator in the AC unit acts as a condenser, and the AC unit coils are dried with warm air. The median relative humidity for all AC units was above 70%, confirming the presence of a favorable environment for microbe growth. In addition, the frequency of appearance of relative humidity values of 90% or higher in homes was 60% in house 006, 74% in house 048, and 58% in house 083, confirming fairly high humidity levels.

### 3.2. Taxonomic Analysis

#### 3.2.1. Taxonomic Identification

Organisms are currently divided into three domains: Eukarya (eukaryotes), Bacteria (eubacteria), and Archaea (archaea). Organisms in each of these domains are further divided by phylum, class, order, family, genus, and species. To allow comparison with previous reports, this study mainly discusses bacteria in terms of phyla and genera.

#### 3.2.2. Phylum

In total, 39 bacterial phyla were detected. The five phyla with the highest mean relative abundance among all samples were *Proteobacteria* (52.21 ± 24.71%), *Firmicutes* (14.19 ± 12.33%), *Actinobacteria* (8.30 ± 6.97%), *Cyanobacteria* (2.15 ± 6.37%), and *Bacteroidetes* (1.50 ± 1.40%) (Figure S1).

#### 3.2.3. Genus

**Figure 4:**
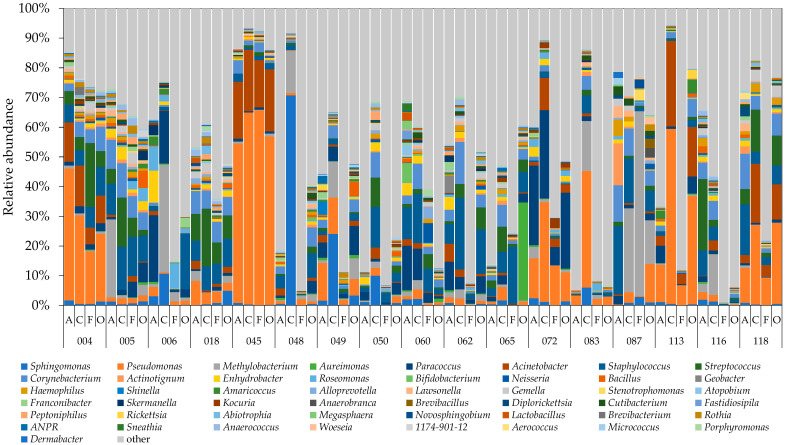
Relative abundance of 2% or higher of bacterial genera for all samples from the 17 houses. A, C, F, and O corresponded to air filter, cooling coil, fan, and air outlet. Genus *ANPR*: *Allorhizobium-Neorhizobium -Pararhizobium-Rhizobium*.

The 10 highest-ranked bacterial genera were *Sphingomonas* (*Proteobacteria* phylum, Gram-negative), *Pseudomonas* (*Proteobacteria* phylum, Gram-negative), *Methylobacterium* (*Proteobacteria* phylum, Gram-negative), *Aureimonas* (*Proteobacteria* phylum, Gram-negative), *Paracoccus* (*Proteobacteria* phylum, Gram-negative), *Acinetobacter* (*Proteobacteria* phylum, Gram-negative), *Staphylococcus*, (*Firmicutes* phylum, Gram-positive), *Streptococcus* (*Firmicutes* phylum, Gram-positive), *Corynebacterium* (*Actinobacteria* phylum, Gram-positive), and *Actinotignum* (*Actinomycetota* phylum, Gram-positive) (Figure 4). The *S. paucimobilis* species, of the genus *Sphingomonas*, is reportedly associated with *pseudobacteraemia*, and cases of severe and invasive infections, such as bacterial arthritis and osteomyelitis, are also not uncommon [38]. The genera *Pseudomonas*, *Methylobacterium*, and *Acinetobacter* are also known to include species that cause opportunistic infections.

The top 10 relative abundance of the mean of each genus were *Pseudomonas*, *Staphylococcus*, *Methylobacterium*, *Paracoccus*, *Acinetobacter*, *Corynebacterium*, *Streptococcus*, *Sphingomonas*, *Enhydrobacter*, and *Roseomonas*—of which 7 were Gram-negative (Figure S2). Furthermore, the median result for the five genera *Pseudomonas*, *Methylobacterium*, *Corynebacterium*, *Streptococcus*, and *Sphingomonas* was higher at the cooling coil than at other locations, showing that many bacteria grow predominantly on the coil surface, which is a high-humidity environment. Using culture-based analysis, Hugenholtz et al. detected *Acinetobacter*, *Corynebacterium*, *Pseudomonas*, and *Staphylococcus* on the coil of an air handling system [19]. Using NGS, Bakker et al. detected predominantly *Methylobacteriaceae*, *Acetobacteraceae*, *Sphingomonas*, *Pseudanabaenaceae*, *Streptophyta*, *Acinetobacter*, and *Corynebacterium* on the surface of an AC unit coil [22].

#### 3.2.4. Change in Ranking of Bacteria Adhering to Various Parts of Air Conditioners

The ten bacterial genera detected with the highest relative abundance on the air filter surface across all 17 residences were analyzed to understand the changes in the relative abundance of bacterial genera in each part of the air conditioner downstream from the air filter. *Pseudomonas* was the most abundant genus at all locations, showing that the bacteria belonging to the genus *Pseudomonas* grow not only in the general environment but also inside AC units. The nine most abundant genera on the air filter matched the nine most abundant genera on the cooling coil and also the seven most abundant genera on the fan and the air outlet, indicating that these bacteria pass through the air filter to adhere to and grow on the cooling coil, fan, and air outlet surface (Table 2). This result is probably due to the generally less-than-excellent collection efficiency of air filters found in room AC units.

#### 3.2.5. Diversity of Bacterial Community

The Shannon index was used to assess alpha diversity. The Shannon index is higher when the number of bacterial species is high and each species is equally present. The order of the highest mean value and the standard deviation of the Shannon index was air outlet (4.858 ± 1.369), air filter (4.725 ± 1.100), cooling coil (4.556 ± 1.077), and fan (4.127 ± 0.816). The bacterial flora on the air outlets was affected both by the indoor environment (when the AC was not in operation) and the AC interior (when the AC was in operation), and the bacterial flora on the air filters was affected by the indoor environment, so the Shannon index was higher. In the air conditioner, the Shannon index was higher on the surface of the condensation coil than on the surface of the fan.

**Figure 5:**
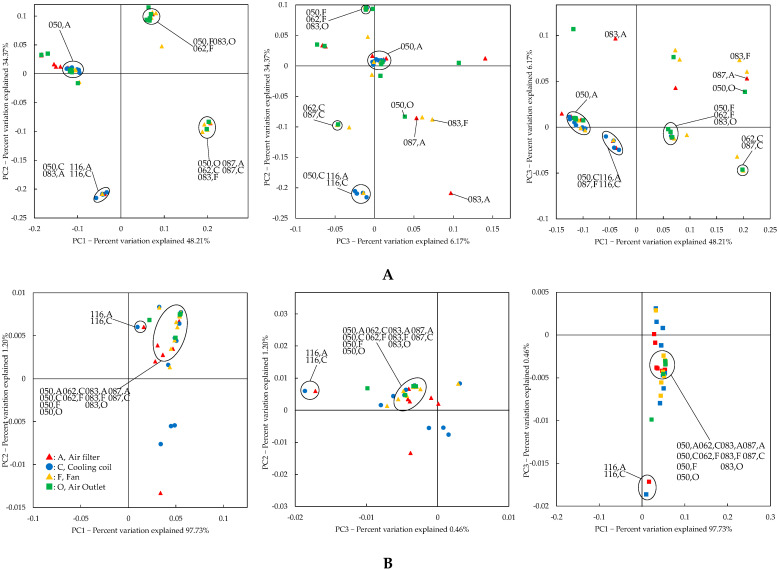
Principal coordinate analysis (PCoA) of partial expansion of the unweighted UniFrac distance (**A**) and weighted UniFrac distance (**B**). A, C, F, and O corresponded to air filter, cooling coil, fan, and air outlet.

Beta diversity is shown by principal coordinate analysis (PCoA) using the unweighted and weighted UniFrac distance [39]. The air filter, the coil, the fan, and the air outlet were plotted close together in house 050; the air filter and the coil were plotted close together in houses 087 and 166; the coil and the fan were plotted close together in house 062; and the air filter, the fan, and the air outlet were plotted close together in house 083 (Figure 5B). AC parts plotted close to each other have similar bacterial flora. However, the flora of the air filter in house 049 and the fan in house 087 was significantly different from that in the other houses: the largest relative abundance of adherent bacteria in the air filter of house 049 was 12.70%, for *Pseudomonas*, and the largest relative abundance of adherent bacteria in the fan in house 087 was 62.20%, for *Methylobacterium* (Figure S3).

## 4. Discussion

Bacterial contamination in air conditioning units affects the health of occupants. The establishment of bacterial flora in air conditioners is influenced by the location of the residence, occupant activity, frequency of air conditioner use, and air conditioner maintenance. Although there have been reports of studies on some parts of air conditioners (filters and coils), there have been almost no reports on the bacterial flora in different components of air conditioners. Therefore, to better understand the bacterial flora inside room AC units, this study sampled bacteria adhering to the air filter, cooling coil, fan, and air outlet of AC units and analyzed the 16S rRNA gene using NGS-based amplicon sequencing. 

The five top-ranked phyla (*Proteobacteria*, *Firmicutes*, *Actinobacteria*, *Cyanobacteria*, and *Bacteroidetes*) were detected in high relative abundance on the filters, coils, fans, and air outlets of all AC units. A study of house dust by Täubel et al. detected *Proteobacteria*, *Firmicutes*, *Actinobacteria*, and *Bacteroidetes* at 1% or greater abundance by sequencing. A study by Dannemiller et al. [34] of bacterial flora in dust collected from the carpets or floors of residential homes detected similarly large amounts of bacteria belonging to the phyla *Firmicutes* and *Actinobacteria*. These findings suggest that these bacterial phyla favor growing in the home environment and appear to pass through the air filter to affect the interior of the AC unit. 

We also investigated the ten highest-ranked bacterial genera adhering to air filters and their subsequent alterations in downstream coils, fans, and air outlets to better understand the characteristics and differences of the bacterial flora in each component of the air conditioner and the differences among them. Because some of the airborne bacteria present at the AC unit air inlet are normally removed by the air filter, if no bacterial proliferation occurs inside the AC unit, the surface of the air filter will contain the largest number of bacteria. Put differently, if the relative abundance of a given bacterium is higher downstream of the air filter (on the coil, etc.), that bacterium probably proliferated at the downstream location. Conversely, because the air outlet of the AC unit is exposed to the air in the room, the indoor environment has a significant impact on the air outlet flora when the AC unit is not in operation. Of the top 10 genera of bacteria detected on the filter surface, 6 genera were Gram-negative (*Pseudomonas*, *Paracoccus*, *Acinetobacter*, *Methylobacterium*, *Enhydrobacter*, and *Sphingomonas*) and 4 genera were Gram-positive (*Staphylococcus*, *Corynebacterium*, *Streptococcus*, and *Actinotignum*). As mentioned above, Gram-negative bacteria have endotoxin in their cell walls, which may lead to allergic sensitization in humans and to workplace-related illnesses. Gram-positive bacteria, however, are known for their cell-wall-associated proteins that play key roles in both colonization and pathogenesis [40]. When an AC unit operates in cooling mode, condensation on the surface of the cooling coil creates a high-humidity environment that favors bacterial proliferation. The seventh-ranked genus on the air filter (*Methylobacterium*) became the second-ranked genus on the cooling coil and the fan, and the ninth-ranked genus on the air filter (*Sphingomonas*) became the third-ranked genus on the cooling coil. Given that *Methylobacterium* bacteria are also detected in bathrooms [41], this genus seems to proliferate readily in high-humidity environments. Bacteria of the genus *Methylobacterium* are also known to form biofilms. The bacterial genera detected in abundance on the cooling coil surface in this study have also been detected in previous studies. Bacteria belonging to the genera *Methylobacteriaceae* and *Sphingomonas* were detected in abundance on AC unit cooling coils by Bakker et al. [22], and bacteria of the genus *Methylobacterium* were detected in abundance on the surface of the heat exchanger (coil) of AC units in four Japanese homes by Hatayama et al. [20]. In the same report, Hatayama et al. also detected an abundance of bacteria of the family *Sphingomonadaceae*, genus *Sphingomonas*. Thus, we can infer that bacteria of the genera *Methylobacterium* and *Sphingomonas* grow readily inside AC units. In this study, bacteria of the genera *Staphylococcus*, *Paracoccus*, and *Streptococcus* were detected with a higher relative abundance on the fan than on the cooling coil. Furthermore, because the AC unit air outlet is affected by the indoor environment when not in operation, the same five bacterial genera were the most abundant on the air filter and on the air outlet. Nevertheless, we did observe some difference in the relative abundance based on location, with the genus *Gemella* ranked in the top 10 on the cooling coil and the fan and the genera *Roseomonas* and *Neisseria* ranked in the top 10 on the fan, but none of these 3 genera ranked among the top 10 on the air filter. The genus *Gemella* is a Gram-positive bacterium that is ubiquitous on the mucous membranes of the human oral cavity, the upper respiratory tract, and the gastro-intestinal tract. Bacteria of the genus *Neisseria* are Gram-negative and, except for *N. gonorrhoeae* and *N. meningitidis*, which are pathogenic, they are endemic in the oral cavity. Bacteria of the genus *Roseomon* are Gram-negative and usually found in the environment.

The PCoA analysis revealed that five residences (050, 062, 083, 087, and 116) had remarkably comparable flora inside the air conditioner, confirming that the same bacterial genera on the surface of the air filter also influence the bacterial contamination inside the air conditioner. However, in many other homes in our study, the bacterial flora inside the air conditioners was not so similar. In other words, the bacterial flora was changing in ACs. Various factors, including the location of the house, ventilation conditions (that is, affected by both indoor and outdoor air [7]), AC unit operating conditions, and occupant activity, make it difficult to quantitatively evaluate the effect of each of these factors on bacterial flora inside the AC unit.

## 5. Conclusions

The AC unit air filter surface provides a history of airborne bacteria in the indoor environment. In our study, of the top 10 genera of bacteria detected on the filter surface, 6 genera were Gram-negative (*Pseudomonas*, *Paracoccus*, *Acinetobacter*, *Methylobacterium*, *Enhydrobacter*, and *Sphingomonas*) and 4 were Gram-positive (*Staphylococcus*, *Corynebacterium*, *Streptococcus*, and *Actinotignum*). Since bacteria on the surface of the air filter affect the bacterial contamination inside the air conditioner, it is important to clean air filters before and during the air conditioning season from the standpoint of occupants’ health. In addition, periodic cleaning of the air conditioner’s internal components is important. However, in many homes in our study, the bacterial flora inside the air conditioners was not so similar. In other words, the bacterial flora was changing. The presence of various impacting factors, including the location of the house (that is, influence from outside air), ventilation conditions, AC unit operating conditions, and occupant activity, makes it difficult to quantitatively evaluate the effect of each of these factors on bacterial flora inside the AC unit. A more detailed investigation of this topic is required.

## Figures and Tables

**Table 1:** Information on the measured residential buildings and room air conditioners.

ID	Building Age	Type of Residental Building		AC Set Point	Frequency of Use	Hours of Opretion	Years of Use	Number of Residents
	(Years)	(Floors)		(°C)	(Day/Week)	(h/day)	(Years)	
004	13	Detached house	1	29	7	12	13	2
005	16	Detached house	1	28	7	24	16	2
006	19	Detached house	1	26	7	17	6	5
018	42	Comdminium	2	26	4.5	12	10	2
045	51	Detached house	1	28	1	2	5	2
048	31	Comdminium	1	27	7	8	6	2
049	44	Detached house	1	26	7	9	10	2
050	45	Detached house	1	26	7	15	8	2
060	11	Detached house	1	23~25	7	12	1.6	2
062	24	Detached house	1	27	7	14	13	2
065	49	Detached house	1	26	7	10	4	2
072	16	Detached house	1	27	7	16	2	3
083	28	Detached house	2	28	7	4	8	4
087	23	Comdminium	13	28	7	24	12	2
113	41	Detached house	1	28	7	5	13	2
116	27	Comdminium	3	26	7	24	10	2
118	7	Detached house	1	26	5	10	7	2

**Table 2:** Change in the ranking of the relative abundances of the top 10 air-filter-adherent bacteria.

	Air Filter		Cooling Coil		Fan		Air Outlet
*Pseudomonas*	1	→	1	→	1	→	1
*Staphylococcus*	2	⤵	4	⤴	3	⤴	2
*Paracoccus*	3	⤵	8	⤴	7	⤴	3
*Corynebacterium*	4	⤵	6	→	6	⤴	5
*Acinetobacter*	5	→	5	→	5	⤴	4
*Streptococcus*	6	⤵	7	⤴	4	⤵	7
*Methylobacterium*	7	⤴	2	→	2	⤵	9
*Enhydrobacter*	8	⤵	9	⤵	16	⤴	12
*Sphingomonas*	9	⤴	3	⤵	17	⤵	11
*Actinotignum*	10	⤵	29	⤴	25	⤴	24

## Data Availability

Not applicable.

## Supplementary Materials

The following supporting information can be downloaded at: https://www.mdpi.com/article/10.3390/microorganisms10112246/s1, Figure S1: Relative abundance of bacterial phyla for all samples from the 17 houses. A, C, F, and O corresponded to air filter, cooling coil, fan, and air outlet; Figure S2: Relative abundances (quartile value) of the main genus. A, C, F, and O corresponded to air filter, cooling coil, fan, and air outlet; Figure S3: Principal coordinate analysis (PCoA) of the unweighted UniFrac distance (A) and weighted UniFrac distance (B).
