# Supplementary material for: Bacterial Communities in Various Parts of Air-Conditioning Units in 17 Japanese Houses
Source: Microorganisms. 2022 Nov 13;10(11):2246. doi: 10.3390/microorganisms10112246 (PMC9697849; doi:10.3390/microorganisms10112246)
Supplement: Supplementary file 1 [file microorganisms-10-02246-s001.zip › microorganisms-2006379-supplementary.pdf]

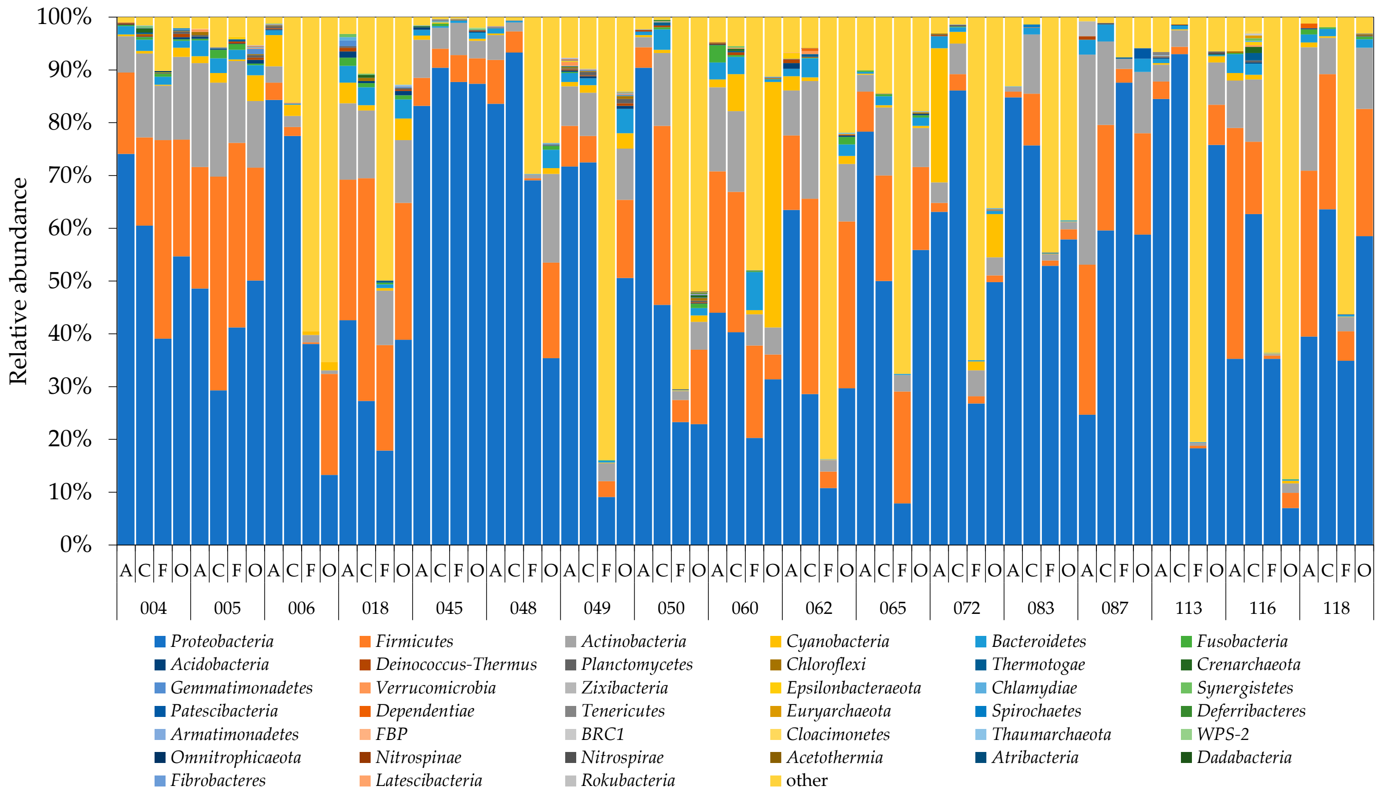

Figure S1. Relative abundance of bacterial phyla for all samples from the 17 houses. A, C, F, and O corresponded to air filter, cooling coil, fan, and air outlet.

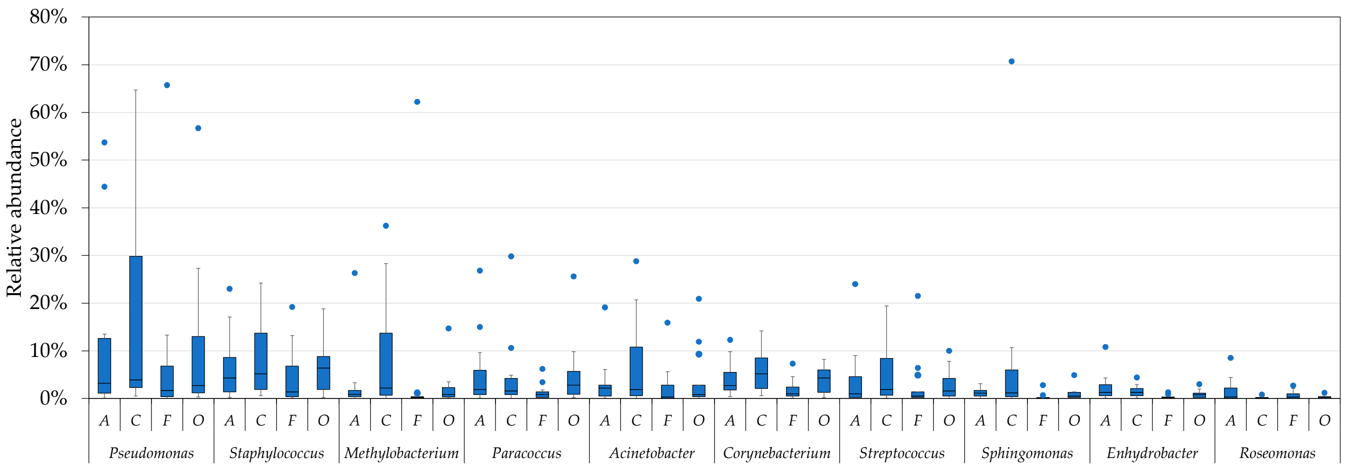

Figure S2. Relative abundances (quartile value) of the main genus. A, C, F, and O corresponded to air filter, cooling coil, fan, and air outlet.

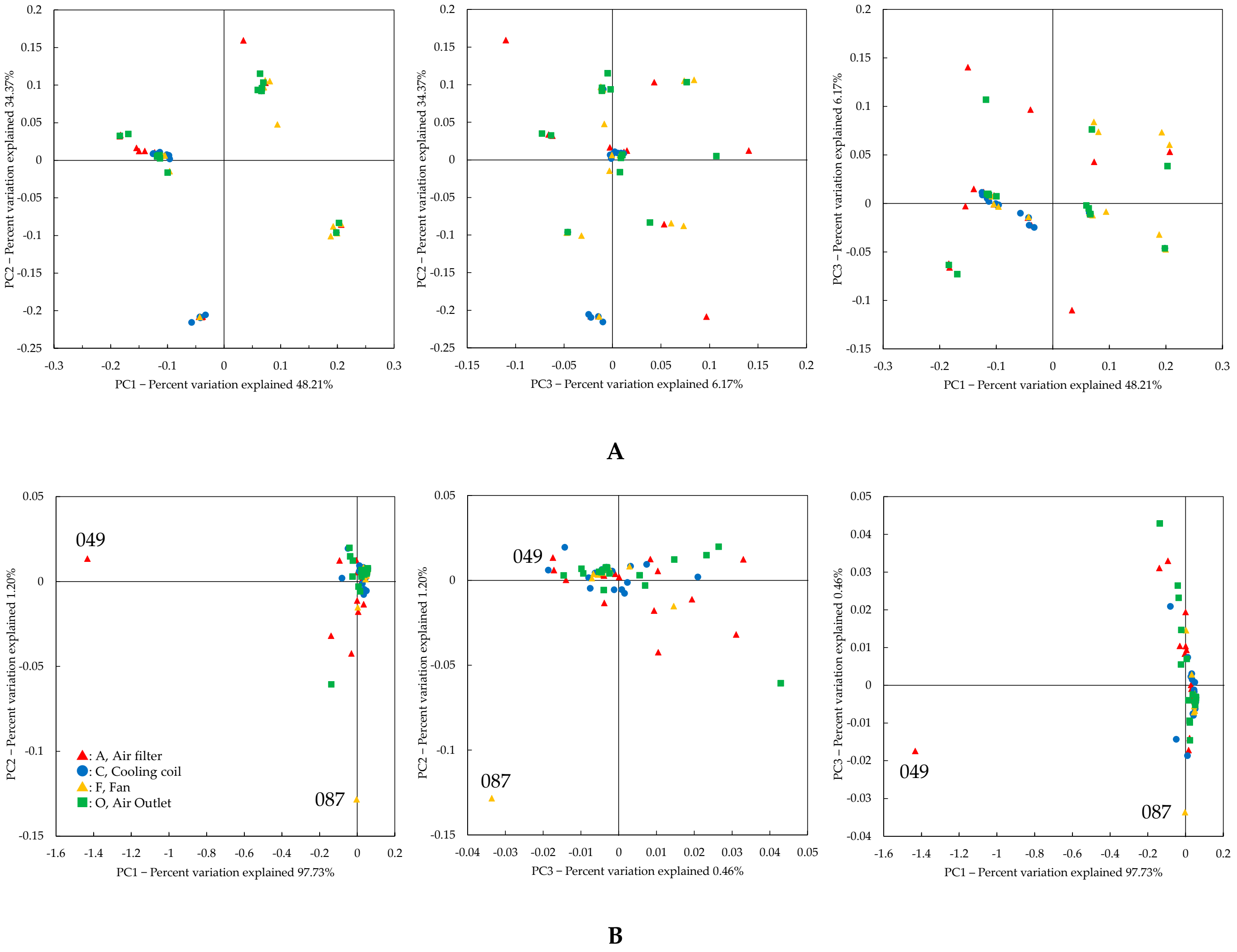

Figure S3. Principal coordinate analysis (PCoA) of the unweighted UniFrac distance (A) and weighted UniFrac distance (B).

A, C, F, and O corresponded to air filter, cooling coil, fan, and air outlet.
